# Supplementary material for: Large-Scale Isolation of Milk Exosomes for Skincare
Source: Pharmaceutics. 2024 Jul 11;16(7):930. doi: 10.3390/pharmaceutics16070930 (PMC11279399; doi:10.3390/pharmaceutics16070930)
Supplement: Supplementary file 1 [file pharmaceutics-16-00930-s001.zip › pharmaceutics-3001956-supplementary.pdf]

Large-scale isolated milk exosomes for skincare

Xue Wu[#], Jiuheng Shen[#], Youxiu Zhong, Xian Zhao, Wantong Zhou, Peifen Gao, Xudong Wang*, Wenlin An*

National Vaccine & Serum Institute (NVSI), China National Biotech Group (CNBG), Sinopharm Group, No. 38 Jing Hai Second Road, Beijing 101111, China

Correspondence:

Xudong Wang, Fax: +86 010 52245226. Email: wangxudong23@sinopharm.com

Wenlin An, Fax: +86 010 52245228. Email: anwenlin@sinopharm.com

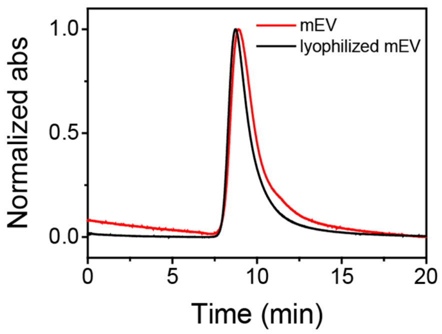

Figure S1. High performance liquid chromatography (HPLC) results of fresh milk exosomes (red) and lyophilized milk exosomes (black).

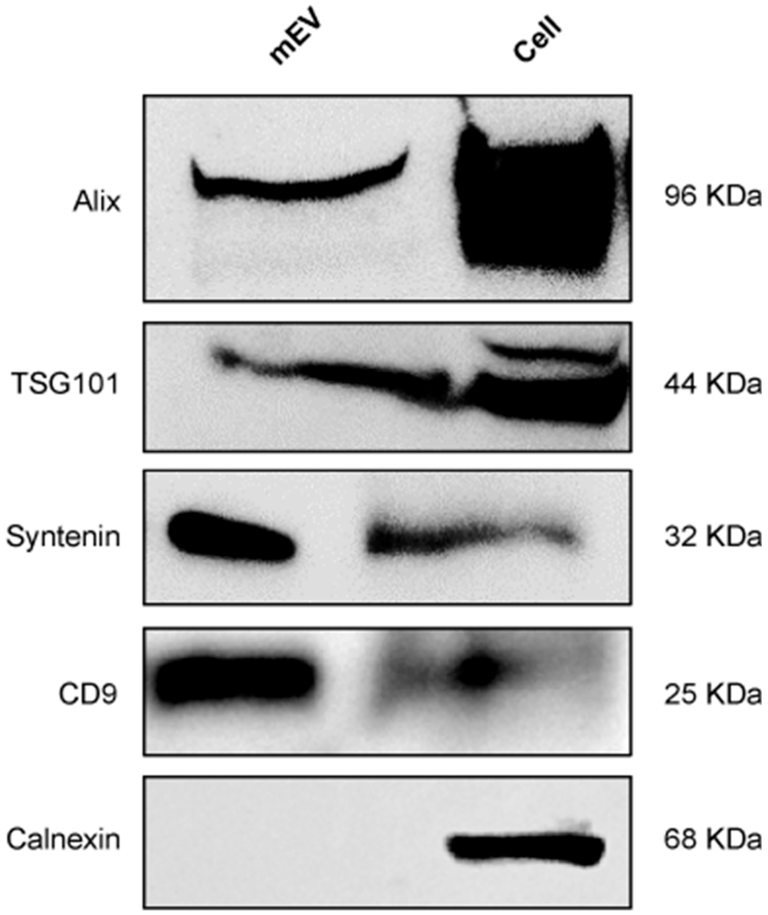

Figure S2. Western blot analysis of EV markers Alix, TSG101, syntenin, CD9 and Calnexin marker of endoplasmic reticulum. The proteins from milk exosomes and cells were extracted and prepared for electrophoresis.

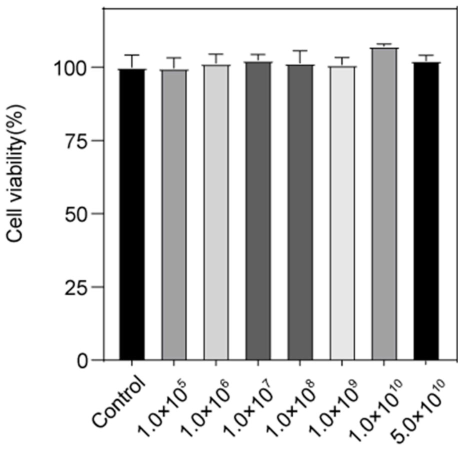

Figure S3. The effect of mEVs ranging from 1.0×10^5 to 5.0×10^{10} particles/ml on the cell viability of NIH3T3 cells tested by CCK-8 assay.

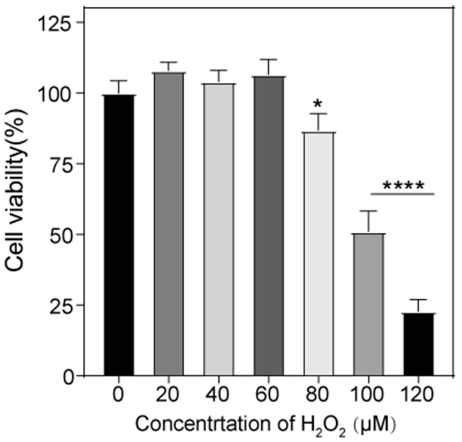

Figure S4. Cell viability toward different concentration H_2O_2 tested by CCK-8 assay.

Table S1. Toxicity parameters of mEVs tested in eggs of zebrafish and teratogenicity of mEVs.

Concentration (g/L)	Mortality (%)	Teratogenicity (%)
0	0	0
0.625	0	0
1.25	0	0
2.5	10	10
5	20	40
10	75	75

Table S2. Multiple skin irritation test of mEVs on New Zealand rabbits.

Days	Animal population	Stimulus response score					
		mEVs			Control		
		Erythema	Edema	Sum	Erythema	Edema	Sum
1	4	0	0	0	0	0	0
2	4	0	0	0	0	0	0
3	4	0	0	0	0	0	0
4	4	0	0	0	0	0	0
5	4	0	0	0	0	0	0
6	4	0	0	0	0	0	0
7	4	0	0	0	0	0	0
8	4	0	0	0	0	0	0
9	4	0	0	0	0	0	0
10	4	0	0	0	0	0	0
11	4	0	0	0	0	0	0
12	4	0	0	0	0	0	0
13	4	0	0	0	0	0	0
14	4	0	0	0	0	0	0
Mean score per animal in 14 days		0.00			0.00		
Mean score per animal daily		0.00			0.00		

Table S3. Acute percutaneous toxicity test of mEVs in rats. (n=10, 5 males and 5 females).

Group	Weight (x±SD) (g)				Poisoning presentation	n (deaths)	Mortality (%)	Abnormality of autopsy
	0d	1d	7d	14d				
Male	200.6±0.47	203.5±1.2	210.9±1.7	219.5±1.3	no	0	0	no
Female	251.1±23.6	255.4±23.5	305.1±13.1	313.0±11.5	no	0	0	no

Table S4. Annotation-List of all identified proteins in milk exosomes (xlsx)

Table S5. Annotation-List of all identified miRNAs in milk exosomes (xlsx)
